# Unsuppressed HIV viral load among people living with HIV by exposure category in British Columbia, Canada from 2005 to 2020: a population-based cohort study

**DOI:** 10.1186/s44263-025-00209-y

**Published:** 2025-10-09

**Authors:** Siu-Kae Yeong, Monica Ye, Rolando Barrios, Mark Hull, Jenny Li, Paul Sereda, Tharit Charoenpanon, Viviane D. Lima, Kirsten Bobrow, Althea Hayden, Rakel Kling, Xinmiao Wang, Nathan Lachowsky, Julio Montaner, David M. Moore

**Affiliations:** 1 Faculty of Medicine, University of British Columbia, Vancouver, BC Canada; 2 British Columbia Centre for Excellence in HIV/AIDS, Vancouver, BC Canada; 3 British Columbia Centre for Disease Control, Vancouver, BC Canada; 4 Vancouver Coastal Health, Vancouver, BC Canada; 5 Northern Health Authority, Prince George, British Columbia Canada; 6 University of Victoria, Victoria, BC Canada

**Keywords:** HIV, MSM, Men who have sex with men, Injection drug use, Community viral load, Suppressed viral load, Unsuppressed viral load, Antiretroviral therapy

## Abstract

**Background:**

Maintaining a suppressed HIV plasma viral load (pVL) is critical to the optimal management of people living with HIV (PLWH), and to prevent HIV transmission. We examined trends and determinants of unsuppressed pVL among PLWH from April 2005 to March 2020 in British Columbia (BC), Canada by HIV exposure groups.

**Methods:**

We analyzed data from all PLWH in BC identified in a provincial database linking testing, treatment, laboratory, pharmacy and administrative health data. For each year, individuals were classified as having an unsuppressed pVL if they were newly diagnosed, had any pVL ≥ 200 copies/mL test result, or had no pVL measurement. We used univariate generalized estimating equations (GEE) to examine trends over time and multivariate GEE modelling to examine factors associated with unsuppressed pVL.

**Results:**

PLWH in BC increased from 5712 in 2005 to 8012 in 2020, and the proportion of those with pVL ≥ 200 copies/mL decreased from 66.4% to 18.1% (p < 0.0001, test of trend). Those who were people who inject drugs (PWID) (adjusted odds ratio [aOR] = 1.92; 95% CI 1.77–2.08) and those with only heterosexual exposures (aOR = 1.16; 95% CI 1.07–1.26) or other exposures (aOR = 1.23; 95% CI 1.06–1.42) had increased odds of unsuppressed pVL, compared with men who have sex with men (MSM). Unsuppressed pVL was also associated with female gender identity (*p* = 0.0109), first CD4 cell count result (*p* = 0.0085), year of ART initiation, health authority residence, hepatitis C seropositivity, and younger age at diagnosis (*p* < 0.001 for all).

**Conclusions:**

The proportion and number of PLWH with unsuppressed pVL in BC fell signficantly over a 15-year period, with different trends by HIV exposure categories. Those who are PWID, females, and youth may require additional supports to be retained in effective HIV care.

## Background

At the end of 2020, an estimated 9,637 people were living with HIV (PLWH) in British Columbia (BC), and an estimated 62,790 people were living with HIV in Canada [1]. Antiretroviral therapy (ART) coverage in BC has increased steadily from approximately 3000 PLWH on treatment in 2003 to over 8000 in 2017 [2]. Starting in 2010, BC formally adopted a program of expanded HIV testing, linkage to care and treatment known as Seek and Treat for Optimal Prevention of HIV/AIDS (STOP HIV/AIDS) [3], which has operationalized the concept of HIV Treatment as Prevention (TasP) [4] as a policy and has been associated with marked declines in HIV-related morbidity and mortality, as well as new HIV infections [5]. As such, the rate of new HIV diagnoses in BC has decreased over time from 7.6 per 100,000 population (335 cases) in 2009 to 2.7 per 100,000 population (137 cases) in 2020 [6], which is at its lowest point since regular reporting started in 1985 [7].

TasP has since been endorsed by the World Health Organization, the United Nations Joint Program on AIDS (UNAIDS) [8] and many other jurisdictions, including the United States Centers for Disease Control and Prevention (US CDC). Furthermore, the United Nations incorporated TasP related targets as the strategy to “End HIV as a pandemic by 2030” within the Sustainable Development Goals [9]. Research has shown that PLWH receiving ART that suppresses their plasma viral load (pVL), to < 200 copies/mL, do not transmit HIV to their sexual partners [10] and are far less likely to transmit HIV during pregnancy, delivery and breastfeeding [11]. HIV treatment and associated virologic suppression have also been shown to contribute to reduced HIV transmission among people who use injection drugs [12]. The US CDC has recommended that measures of engagement in HIV treatment and virologic suppression, such as community pVL, be included as additional surveillance tools for monitoring HIV epidemics. The US CDC defines community pVL as “a population-based measure of HIV-infected individuals’ concentration of plasma HIV-1 RNA” [13]. Several studies have shown correlations between community pVL and declines in new HIV diagnoses [14–16].

The prevention benefits of HIV treatment are evident at the population level. In BC, mathematical models have demonstrated that every 1% increase in the number of people with suppressed virus on ART was associated with a 1.2% reduction in estimated HIV incidence [5]. Other jurisdictions, including Los Angeles [17], New York [18], the United Kingdom [19], Sweden [20], and India [21], have demonstrated disparities among sub-groups of PLWH in terms of community pVL and found several factors associated with unsuppressed pVL including gender, substance use and HIV exposure category. We have seen variations in the trajectory of the HIV diagnoses in BC by exposure category [22] and wanted to examine how this mapped onto trends in virologic suppression among PLWH. In this paper, we examined trends in treatment status for PLWH in BC from April 2005 to March 2020 to see whether engagement in care has significantly affected the proportions and absolute numbers of those with unsuppressed pVL over time. We further examined factors associated with having a pVL measurement ≥ 200 copies/mL (or no pVL measurement) each year among PLWH, with a particular interest in examining variations by HIV exposure category.

## Methods

### Participants and data collection

We conducted a retrospective cohort analysis, including all PLWH in BC from April 2005 to March 2020, identified in the provincial STOP HIV/AIDS database; this database has been previously described elsewhere [23–25]. It includes data from the BC HIV/AIDS Surveillance System [26], the BC HIV Drug Treatment Program (DTP) [27, 28], the BC PharmaNet database [29] and health service utilization data through physician billing data from the provincial Medical Services Plan (MSP) [30, 31] and the provincial Hospital Discharge Abstract Database [32] and linkages to the BC Vital Statistics registry [33]. In BC, ART is provided to all medically-eligible individuals living with HIV through the HIV Drug Treatment Program of the BC Centre for Excellence in HIV (BC CfE) at no cost to patients following a prescription from any physician in the province. BC has five regional health authorities that govern, plan and deliver health-care services within their geographic areas. The Health Authorities with the largest populations are Vancouver Coastal Health, which includes the city of Vancouver and bordering suburbs, and Fraser Health Authority, which largely occupies suburban Vancouver.

Information on the HIV testing component includes data on approximately 95% of all screening and all confirmatory HIV testing done in the province [24]. The database also contains information on all ART dispensing, pVL and CD4 cell counts. All HIV pVL testing in the province is conducted centrally at the St Paul’s Hospital virology laboratory in Vancouver, under the auspices of the BC CfE which ensures we have full capture of these data. Similarly, the BC CfE is responsible for the procurement and fully subsidized distribution of all antiretroviral medications to all PLWH in the province, which ensures we have full capture of these data.

Participants who were known to be alive and residing in BC as of April 2005, or who were diagnosed or living with HIV or moved to BC from April 2005 to March 2020 were included in the analysis. Note that the data provided by the Ministry of Health was organized on the basis of fiscal year (April–March) rather than calendar year.

### Data analysis

For each year starting in April 2005, individuals were classified as having an unsuppressed pVL if they met the following criteria: 1) were newly diagnosed; 2) had any pVL ≥ 200 copies/mL measure; or 3) did not have any pVL measure. Individuals living with HIV who were known to have moved out of the province or who had died were censored at the date of migration or death. Additionally, participants were censored if more than two years passed without any record of CD4, pVL, ART prescription or any other MSP billable visit or hospitalization and Vital Statistics linkages did not identify them as having died. Such individuals were assumed to have emigrated from the province without notifying the program. MSP records were used to generate time-updated health authority residence data. We used univariate generalized estimating equations (GEE), with year as the explanatory variable to examine trends over time and multivariate GEE modelling to examine factors associated with unsuppressed pVL. The multivariable logistic regression model, again using GEE, examined demographic and clinical factors associated with having at least one unsuppressed pVL or no pVL measurement.

The primary outcome was unsuppressed pVL (HIV pVL ≥ 200 copies/mL) or no pVL in a given calendar year. The following explanatory variables were examined for potential inclusion in the multivariate model: age at diagnosis, year of ART start, HIV exposure category (men who have sex with men (MSM) (including MSM who also use injection drugs), people who use injection drugs [PWID], heterosexual only, or other), hepatitis C Virus (HCV) serology, CD4 cell count at baseline, and health authority of residence. Gender identity was constructed from gender recorded on DTP enrollment forms or prescription refills, as well as sex recorded in other health records. Transgender individuals included those who identified their gender identity as transgender as well as those whose gender identity was different from their sex at birth.

We estimated the associations between the primary outcome of unsuppressed viral load and the covariates, with a p-value of < 0.05 as the threshold for statistical significance. Variables with > 50% missing data were not considered for inclusion in multivariate models and missing values were included as specific category for each variable. Variable categories with relatively small sample sizes were combined to provide more reliable estimates. We tested for collinearity using the Variance Inflation Factor (VIF). For categorical variables, we first converted them into dummy variables, and used VIF to assess potential multicollinearity arising from redundancy within these categories or correlations with other explanatory variables. For continuous variables, VIF was used to determine whether any explanatory variable can be explained by the others in the model. Reference groups were selected as the largest group for each variable. The model assumes a linear relationship between each continuous explanatory variable and the population-averaged log odds of the outcome. We assessed this by plotting Pearson residuals against continuous explanatory variables. All analyses were conducted using SAS version 9.3 (SAS Corporation, Cary, NC).

## Results

We identified 5712 people living with HIV in the STOP HIV/AIDS database in 2005–06, and by 2019–20, this number had increased to 8012. A total of 11 086 unique individuals were included in our analysis. Of these, 1993 (18.0%) died over the study period, and 1046 (9.4%) were censored after more than two years without any record of an interaction with the health care system and did not reappear in the database. Another 186 (1.7%) had no contact with the healthcare system of less than two years but had an unknown status at the end of follow-up. Table 1 shows the demographic and clinical/behavioural characteristics of participants at their first recorded entry in the STOP HIV database.

**Table 1:** Descriptive statistics of 11 086 people living with HIV in British Columbia identified through the STOP-HIV database 2005–2020

Characteristics	n	%
**Gender**		
Male	8400	75.8
Female	1856	16.7
Transgender	42	0.4
Missing data	788	7.1
**HIV exposure category**		
MSM	4703	42.4
Hx of IDU	2863	25.8
Heterosexual	1507	13.6
Other*	447	4.0
Missing	1566	14.1
**ART start era**		
≤ 2000	2170	19.6
2001–2005	1568	14.1
2006–2010	2532	22.8
2011–2015	2871	25.9
2016–2020	1156	10.4
Missing data or never on ART	789	7.1
**Born in Canada**		
No	442	4.0
Yes	2212	20.0
Missing	8432	76.1
**Health authority at diagnosis**		
VCH: city of Vancouver	5	47.1
VCH: other than Vancouver	628	5.7
Fraser	2421	21.8
Vancouver Island	1251	11.3
Interior	658	5.9
Northern	437	3.9
Missing outside of BC	474	4.3
**Hepatitis C antibody positive**		
No	6200	55.9
Yes	3471	31.3
Missing	1415	12.8

^*^Other exposure categories included mother-to child-transmission or blood transfusion

Of the 11 086 PLWH, 8400 (75.8%) identified as male, 1856 (16.7%) identified as female, 42 (0.4%) identified as transgender and 788 (7.1%) had incomplete data on current gender identity. The median age of diagnosis was 37 years (1st quartile (Q1)-3rd quartile (Q3) 30–45 years). In terms of HIV exposure groups, 4703 (42.4%) were MSM, 2863 (25.8%) had a history of IDU and were not MSM, 1507 (13.6%) had only heterosexual exposures, and 447 (4.0%) had other exposures, such as blood transfusion or mother to child transmission. An additional 14.1% had missing HIV exposure information. A total of 42% had missing data on the place of residence at diagnosis. However, among those whose residence was known, the majority of those with residence information at diagnosis resided within Vancouver Coastal Health. The median CD4 count when first recorded was 380 cells/µL (Q1-Q3 220–580 cells/µL). Of the 9,671 participants with laboratory records for HCV serostatus, 31.3% were antibody-positive for HCV, with another 1415 (12.8%) having no record of HCV testing.

**Fig. 1:**
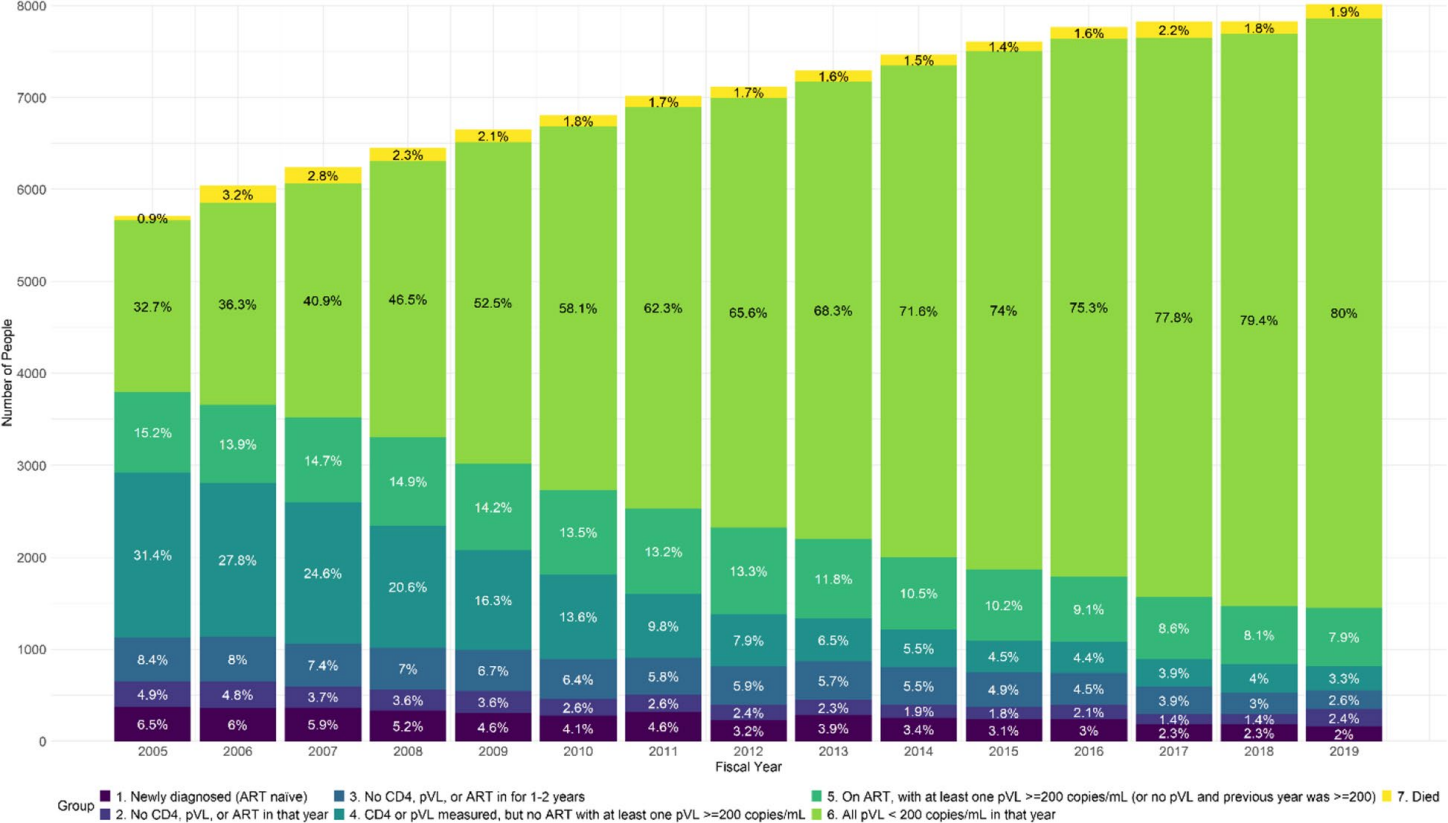
Participants in the British Columbia HIV Drug Treatment Program and treatment, laboratory testing and virologic suppression status: April 2005–March 2020. Note: Years are April of listed year to March of following year

Overall, the proportion of those with unsuppressed pVL decreased from 66.5% in 2005–06 to 18.1% in 2019–20 (p < 0.0001, GEE test of trend) (Fig. 1). This corresponded to a decline in absolute numbers from 3794 PLWH at the end of 2005 to 1452 in 2019. As well, the proportion of those with no VL measured in the year declined from 4.9% to 2.4% and those with no VL or CD4 measured within one to years declined from 8.4% to 2.6%. During the same period, the proportion of those receiving ART increased from 2736/5712 (47.9%) in 2005 to 7045/8012 (87.9%) in 2020 (p < 0.001 for test of trend).

Among MSM, those with unsuppressed pVL declined from 60.0% in 2005 to 13.8% in 2019 (p < 0.0001 test of trend). Among PWID, unsuppressed pVL declined from 76.3% in 2005 to 29.5% in 2019 (p < 0.0001 trend test) and among heterosexuals, those with unsuppressed pVL declined from 61.6% in 2005 to 13.6% in 2020 (p < 0.0001).

In the multivariate model (Table 2), those with a history of IDU (adjusted odds ratio [aOR] = 1.92; 95% confidence interval [CI] 1.77–2.08) and those with only heterosexual exposures (aOR = 1.16; 95% CI 1.07–1.26) had higher odds of unsuppressed pVL, compared with MSM. As well, we found that having an unsuppressed pVL was associated with a younger age at diagnosis (aOR = 0.82 per 10-year increase; 95% CI 0.80–0.84), female gender (aOR = 1.11; 95% Cl 1.03–1.19), and lifetime HCV seropositivity (aOR = 1.38; 95% CI 1.29–1.48). Unsuppressed pVL was also associated with the year of ART initiation, whereby those who initiated ART in 2001–2005 had lower odds of unsuppressed pVL (aOR = 0.89; 95% CI 0.81–0.98) compared with those started on ART in or before the year 2000. Conversely, those started ART in 2006–2010 (aOR = 1.54; 95% CI 1.43–1.65), those started in 2011–2016 (aOR = 1.96; 95% CI 1.82–2.12), and those started ART in 2017–2020 (aOR = 3.31; 95% Cl 2.96–3.69) had higher odds of unsuppressed pVL compared with those started on ART in or before the year 2000. We also found PLWH in Fraser Health Authority (suburban Metro Vancouver) (aOR = 0.86, 95% Cl 0.81–0.91), Vancouver Island (aOR = 0.87, 95% Cl 0.80–0.95) and Interior BC (aOR = 0.89, 95% Cl 0.81–0.99) had lower odds of unsuppressed pVL compared with people living in the city of Vancouver. When testing for collinearity of explanatory variables, all VIF values were below 10, indicating no serious multicollinearity.”

**Table 2:** Generalized Estimating Equations of factors associated with at least one VL ≥ 200 copies/mL or no pVL measured in a year: 2005–2020

	**Multivariable model**			
	**Adjusted odds ratio**	**95% confidence interval**		**p-values**
**Gender**				
Male [ref]	1.00			**0.0109**
Female	1.11	1.03	1.19	
Incomplete data or transgender	0.77	0.52	1.15	
**HIV exposure category**				
MSM [ref]	1.00			** < 0.0001**
PWID	1.92	1.77	2.08	
Heterosexual	1.16	1.07	1.26	
Other*	1.23	1.06	1.42	
Missing	0.74	0.68	0.81	
**ART start year**				
≤ 2000 [ref]	1.00			** < 0.0001**
2001–2005	0.89	0.81	0.98	
2006–2010	1.54	1.43	1.65	
2011–2015	1.96	1.82	2.12	
2016–2020	3.31	2.96	3.69	
Missing or never on ART	10.49	6.45	17.07	
**Current health authority residence**				
VCH: Vancouver [ref]	1.00			** < 0.0001**
VCH: other than city of Vancouver	0.87	0.78	0.98	
Fraser	0.86	0.81	0.91	
Vancouver Island	0.87	0.80	0.95	
Interior	0.89	0.81	0.99	
Northern	1.09	0.94	1.26	
Missing	8.19	7.28	9.21	
**Hepatitis C antibody positive**				
No [ref]	1.00			** < 0.0001**
Yes	1.38	1.29	1.48	
Missing	1.42	1.25	1.62	
**Age at diagnosis (per 10 years)**	0.82	0.80	0.84	** < 0.0001**
**First recorded CD4 cell count (per 100 cells/µL)**	0.99	0.98	1.00	**0.0085**

^*^Other exposure categories included mother-to child-transmission or blood transfusion

## Discussion

Our results show that the proportion and numbers of PLWH with unsuppressed pVL or no pVL measurements have fallen dramatically as the proportion and numbers of those receiving ART have increased in BC between April 2005 and March 2020. During this period, BC adopted increasingly liberalized HIV treatment guidelines and, in 2010, formally implemented TasP as an HIV control strategy [3]. In the last year of this analysis, 88% of people diagnosed with HIV were receiving ART, and 91% of these had a pVL consistently < 200 copies/mL. However, not only are more PLWH accessing treatment in BC, there also appears to be better linkage to care overall, in that fewer participants had missing VL or CD4 measurements in later years.

In the multivariate analysis, we found that PWID had higher odds, 1.92, of unsuppressed pVL compared with MSM. Previous research has found that PWID experience significant structural and systemic barriers to HIV treatment, stigmatization by healthcare providers, and greater delays in linking to HIV treatment, which make it more difficult to engage and remain in care [34, 35]. PWID are also less likely to be prescribed ART, less likely to be retained in care, and once on ART, are less likely to be virally suppressed [36].

We also found that those with only heterosexual exposures to HIV had increased odds (1.16) of unsuppressed pVL compared with MSM. A recent study from the neighbouring province of Alberta also found that episodes of unsuppressed pVL were more common among PWID and PLWH with only heterosexual exposures compared with MSM [37]. Compared with MSM and PWID, there are far fewer PLWH in BC with only heterosexual exposures, and they appear to be a heterogeneous group. An analysis of individuals diagnosed with HIV with only heterosexual exposures from 2009 to 2015 showed that 48% reported having a sexual partner who was living with HIV, 18% had other high-risk contacts (not defined), 13% were born or had resided in a high HIV prevalence country, 11% reported sharing drug paraphernalia, 5% reported engaging in sex work and 2% had a recent STI [38].

We found some variation in pVL suppression by regional health authority, with lower odds of unsuppressed pVL in most health authorities compared with PLWH residing in Vancouver. This likely reflects a larger population of unstably housed individuals, those with substance use and mental health concerns in Vancouver, and the in-migration of individuals seeking services for these conditions.

The proportion of PLWH with suppressed pVL (80%) in the final year of this analysis was higher than what has been reported in many jurisdictions. In an analysis of 2014 data from 33 jurisdictions in the United States from the HIV/AIDS Surveillance System, only 57.3% of people diagnosed with HIV had a suppressed pVL at their last measurement, and 47.6% had sustained virologic suppression for all measurements in a year [39]. By comparison, our data in Fig. 1 demonstrate that 72% of PLWH and diagnosed in BC achieved sustained virologic suppression in 2014. The US CDC estimated that 66% of PLWH in the United States in 2021 were virologically suppressed [40], while we found that 80% were consistently suppressed in 2020. A report from the Ontario (Canada) HIV Epidemiology and Surveillance Initiative also estimated that a similarly high proportion of people who have been diagnosed with HIV, 84.7% were virologically suppressed in 2020 [41]. Both studies used data based only on those who have been diagnosed with HIV although it is not clear whether the latter was adjusted for potentially missing VL data for those not in care. The Public Health Agency of Canada estimates that 6% of people with HIV in BC in 2020 were undiagnosed [1]. However, given the large expansion in HIV testing in BC since 2010 [42], this percentage is likely smaller.

Our analysis also found that having an unsuppressed pVL was associated with younger age at diagnosis. These findings are also consistent with studies from the United States, where unsuppressed pVL among HIV patients has been shown to be significantly associated with younger age at diagnosis, as well as being treatment-inexperienced and a history of injection drug use [43–45]. Hypothesized reasons proposed for these observations include HIV-related stigma and discrimination, social and economic marginalization and unstable housing, among other factors commonly recognized as the social determinants of health [46]. Older age may also be associated with increased recognition of mortality, hence a greater motivation to follow treatment recommendations from health care providers or, by a survivor effect where individuals who maintain greater compliance with treatment recommendations may actually outlive those less adherent [47].

We also found that having an unsuppressed pVL was associated with testing seropositive for HCV. As a consequence of shared routes of transmission, between 20–30% of people living with HIV in Canada are co-infected with the HCV [48], similar to what we found in this study for BC only. Ongoing alcohol and drug use and decreased ART adherence among co-infected persons may also result in increased HIV viral load [49]. HCV/HIV coinfection has also been found to be associated with earlier virologic failure, lower CD4 cell count increases, and increased risk of developing AIDS and mortality [50].

Although TasP appears to be effective across all exposure categories, the trends in new HIV diagnoses in BC do not closely parallel the reductions in unsuppressed pVL we have observed. While we have seen the largest reductions in unsuppressed pVL among MSM living with HIV, new diagnoses among this group continue to comprise the greatest number of new HIV diagnoses in BC [51]. This likely reflects a central tenant of infectious disease epidemiology that prevalence drives incidence. The most recent estimate for the prevalence of HIV among MSM in Metro Vancouver was 20.4% in 2019 [52]. However, the number of new HIV diagnoses among MSM has decreased from 181 [53] in 2005 to 58 in 2020 [6], a 68% reduction. In addition, the number of diagnoses of other sexually transmitted infections in BC, in particular, syphilis, has been steadily increasing over time [54], suggesting that increases in risk behaviour may have limited some of the benefits of TasP in BC. As well, the implementation of a fully publicly-funded province-wide HIV PrEP program, beginning in 2018, has further contributed to reductions in HIV diagnoses among MSM in BC [55].

Somewhat counterintuitively, we have seen the largest decline in new diagnoses, 79.2%, among PWID, which had the smallest reductions in unsuppressed pVL. The number of new HIV diagnoses in BC among PWID decreased from 125 in 2005 [53] to 26 in 2020 [6]. This suggests that additional public health measures beyond TasP have likely contributed to this decrease in new diagnoses among PWID. Over this period, provincial harm reduction programs, such as needle distribution programs, supervised injection sites, and other prevention programs, have dramatically expanded, which has likely contributed to declines in HIV transmission among PWID [56, 57]. Survey data among people who use drugs in Vancouver and Victoria also demonstrated changes in drug use behaviour during this period, from injecting to smoking illicit substances [56].

Our study has several strengths as well as limitations. The STOP HIV/AIDS database includes several data sources in BC, which together can give us a better picture of the HIV epidemic in the province. In particular, it allows us to follow individuals who may be diagnosed but not receiving HIV care and can systematically record the deaths of individuals who are residing in the province even if they are not engaged in any health care. The laboratory database includes all confirmatory HIV tests conducted in BC and anyone who has had a pVL test or has been dispensed ART in BC through the public program. It also includes individuals who do not meet these criteria but are identified through algorithms applied to administrative health records [24]. We had some variables with greater than 50% missing values, which meant that we had a limited number of variables that we could potentially include in the multivariate modelling. We also had many variables with less than 50% missing data, which were included in the models with a separate category for missing. This may have introduced biases into our analyses but the impact and directionality of these biases are unknown. Furthermore, we did not account for migration within BC over time which may account for the differences between health authorities as some individuals from smaller communities. Several factors of interest are not available in the database (e.g. ethno-racial identity), which may help explain patterns of health disparities.

## Conclusions

BC has made great progress in engaging PLWH in HIV care and treatment as evidenced by the decreasing proportion of PLWH with unsuppressed pVL over a 15-year period. However, patients who are younger, female, are PWID, or have tested antibody positive for HCV, may require additional support to engage in and remain on treatment. Our findings demonstrate that a comprehensive provincial HIV control strategy, based on TasP principles, can improve HIV-related outcomes and thus may inform implementation efforts in other provinces across Canada and in other countries. However, additional interventions for specific key populations are also needed in order to make further progress towards the achievement of the UNAIDS treatment targets to end the global HIV/AIDS epidemic.

## Data Availability

The datasets generated and/or analysed during the current study are not publicly available due to provisions in British Columbia Centre for Excellence (BC-CfE) institutional policies, service contracts, and ethics requirements. In order to facilitate research, we make such data available via data access requests. Some BC-CfE data is not available externally due to prohibitions in service contracts with our funders or data providers. For more information or to make a request, please contact Mark Helberg, Senior Director, Internal and External Relations, and Strategic Development: mhelberg@bccfe.ca. All data related to this manuscript are provided in the main body of the paper.

## Abbreviations

ART: Antiretroviral therapy
BC: British Columbia
BC CfE: BC Centre for Excellence in HIV
DTP: Drug Treatment Program
GEE: Generalized estimating equations
HCV: Hepatitis C virus
MSM: Men who have sex with men
MSP: Medical Services Plan
PLWH: People living with HIV
PWID: People who use injection drugs
pVL: Plasma viral load
REC: Research Ethics Committee
STOP HIV/AIDS: Seek and Treat for Optimal Prevention of HIV/AIDS
TasP: Treatment as Prevention
UNAIDS: United Nations Joint Program on AIDS
US CDC: United States Centers for Disease Control and Prevention
